# Comparison Between the Human-Sourced Ellipsoid Method and Kidney Volumetry Using Artificial Intelligence in Polycystic Kidney Disease

**DOI:** 10.3390/jpm15080392

**Published:** 2025-08-20

**Authors:** Jihyun Yang, Young Rae Lee, Young Youl Hyun, Hyun Jung Kim, Tae Young Shin, Kyu-Beck Lee

**Affiliations:** 1 Division of Nephrology and Hypertension, Department of Internal Medicine, Kangbuk Samsung Hospital, Sungkyunkwan University School of Medicine, Seoul 03181, Republic of Korea; jihyun.yang@samsung.com (J.Y.);; 2 Department of Radiology, Kangbuk Samsung Hospital, Sungkyunkwan University School of Medicine, Seoul 03181, Republic of Korea; 3 Department of Urology, Ewha Womans University Mokdong Hospital, Seoul 07985, Republic of Korea; 4 Synergy A.I. Co., Ltd., Seoul 07573, Republic of Korea

**Keywords:** polycystic kidney disease, MCI, total kidney volume, ADPKD, AI-based volumetry

## Abstract

**Background:** The Mayo imaging classification (MIC) for polycystic kidney disease (PKD) is a crucial basis for clinical treatment decisions; however, the volumetric assessment for its evaluation remains tedious and inaccurate. While the ellipsoid method for measuring the total kidney volume (TKV) in patients with PKD provides a practical TKV estimation using computed tomography (CT), its inconsistency and inaccuracy are limitations, highlighting the need for improved, accessible techniques in real-world clinics. **Methods:** We compared manual ellipsoid and artificial intelligence (AI)-based kidney volumetry methods using a convolutional neural network-based segmentation model (3D Dynamic U-Net) for measuring the TKV by assessing 32 patients with PKD in a single tertiary hospital. **Results:** The median age and average TKV were 56 years and 1200.24 mL, respectively. Most of the patients were allocated to Mayo Clinic classifications 1B and 1C using the ellipsoid method, similar to the AI volumetry classification. AI volumetry outperformed the ellipsoid method with highly correlated scores (AI vs. nephrology professor ICC: r = 0.991, 95% confidence interval (CI) = 0.9780–0.9948, *p* < 0.01; AI vs. trained clinician ICC: r = 0.983, 95% CI = 0.9608–0.9907, *p* < 0.01). The Bland–Altman plot also showed that the mean differences between professor and AI volumetry were statistically insignificant (mean difference 159.5 mL, 95% CI = 11.8368–330.7817, *p* = 0.07). **Conclusions:** AI-based kidney volumetry demonstrates strong agreement with expert manual measurements and offers a reliable, labor-efficient alternative for TKV assessment in clinical practice. It is helpful and essential for managing PKD and optimizing therapeutic outcomes.

## 1. Introduction

Autosomal dominant polycystic kidney disease (ADPKD) is the most prevalent inherited cystic kidney disorder, characterized by the progressive formation of renal cysts and various extrarenal manifestations. It occurs in all races, and its prevalence ranges from 1:400 to 1:1000 [1]. A recent study reported the incidence is higher in Black (73 per 100,000), non-Hispanic White (63.2 per 100,000), Hispanic (39.9 per 100,000), and Asian (48.9 per 100,000) individuals [2]. The ADPKD is a complex, genetically inherited disease; the most well-known mutations are PKD 1, PKD 2, and GANAB [2,3,4]. According to the ICD-10 code, the prevalence in South Korea is 1:10,000; however, this value is underestimated because of the limitations of the reported system (low sensitivity and specificity) [5]. ADPKD typically progresses to kidney failure, often requiring dialysis at a relatively young age. [6]. ADPKD is characterized by various clinical presentations. Notably, the rate at which the disease progresses to end-stage kidney disease exhibits substantial variability among affected individuals. This heterogeneity in disease trajectory poses significant challenges in predicting individual outcomes and tailoring effective management strategies. A breakthrough in this regard has been the development of tolvaptan, a vasopressin 2 receptor antagonist designed to suppress the growth of kidney cysts. Recent treatment goals focus on disease modification, delaying kidney failure, and preserving renal function through proactive interventions. Tolvaptan holds significant promise for patients with ADPKD by reducing their dialysis-dependent period.

Radiological findings are crucial in diagnostic algorithms and treatment plans [4,7,8,9,10]. In adults with incidentally discovered multiple renal or hepatic cysts, determining whether the imaging findings are typical or atypical is vital. If there is a family history, ADPKD can be diagnosed definitively; however, in cases of no family history or unclear radiological findings, renal image study, including ultrasonography, computed tomography (CT), or magnetic resonance imaging (MRI), is recommended. In conjunction with assessing the kidney size relative to the height using the Mayo Image Classification (MIC), genetic testing should be considered. The genetic testing is important not only for diagnostic certainty in sporadic or atypical cases but also for confirming eligibility for disease-modifying treatment. If the estimated glomerular filtration rate exceeds 25 mL/min/1.73 m^2^ and rapidly progressive disease is suspected, tolvaptan treatment is recommended. The determination of rapidly progressive ADPKD is based on clinical consensus. As genetic testing is not always feasible, renal imaging becomes a crucial, accessible evaluation method. Patients with a high risk for rapid disease progression, as determined by their height-adjusted total kidney volume (htTKV), are prioritized for tolvaptan therapy.

Despite its promise, the use of tolvaptan as a therapeutic agent presents significant challenges. Several clinical guidelines have been established to identify patients who are most likely to benefit from the medication. Guidelines emphasize the significance of accurately assessing the balance of risks and benefits before initiating treatment, with kidney volume recognized as a critical predictor of disease progression. To define rapid disease progression, the MIC can help guide patients and clinicians. MIC stratifies patients into five groups (1A to 1E) based on age-adjusted height-adjusted total kidney volume. MIC 1 C to 1E would have potential for rapid progressive disease, so the disease-modifying treatment should be considered. However, the accurate measurement of kidney volume remains challenging in clinical practice. MRI is recommended as the first kidney imaging approach; CT and ultrasound are other options [9,11,12]. The selection of an imaging modality is the starting point, as each method has distinct advantages and disadvantages. Clinicians should be trained and informed regarding volume measuring. To address this gap, researchers have developed a semi-quantitative method known as MIC. MIC offers a practical alternative for estimating kidney volume using basic anatomical dimensions—width, depth, and longitudinal length—obtained from MRI or CT [13]. Sharma et al. emphasized the significance of expert operators who can perform a reliable estimation of kidney volume [14]. Sophisticated software that enables stereostatic kidney measurement is reported for better, more precise TKV assessment [15]. While several post-processing software tools can generate kidney segmentation and volume measurements (ITK-SNAP, TotalSegmentator, Synopsys Simpleware, Phillips IntelliSpace, Siemens Syngo.via), their adoption remains inconsistent across institutions, primarily due to limited standardization and insufficient user training.

While the MIC represents a valuable tool for estimating kidney volume in patients with ADPKD, its limitations highlight the need for continued research and innovation in this field. Accurate TKV measurement remains central to ADPKD management, representing a key factor in determining disease progression and guiding therapeutic decisions. However, the process is time-consuming and not error-free. New measurement methods that offer increased speed, accuracy, and user convenience are highly essential [11,16,17,18]. Shin et al. reported an artificial intelligence (AI)-based decision-supporting system in tolvaptan therapy called Ignite™ [19]. Therefore, we aim to compare and evaluate AI-based htTKV measurement and ellipsoid method-derived kidney size values, as well as assess their MIC similarities.

## 2. Methods

### 2.1. Data Collection

We retrospectively collected imaging data of patients with ADPKD who underwent MRI and CT within 6 months between January 2015 and March 2024. Patients with typical ADPKD, regardless of maintaining tolvaptan therapy, were reviewed. The 6-month interval was chosen considering that kidney size would not exhibit significant growth within this period. We aimed to evaluate the agreements between kidney size measurements obtained using the MIC by expert (nephrology professor), trained non-expert (trained clinician), and AI-based kidney volumetry (Ignite™, Synergy A.I. Co., Seoul, Republic of Korea) [19]. Our TKV calculation algorithm uses a semantic segmentation model, 3D Dynamic U-Net, to identify ADPKD regions from abdominopelvic CT scans. The model is trained on paired 3D CT images and corresponding mask arrays to learn spatial features.

**Figure 1:**
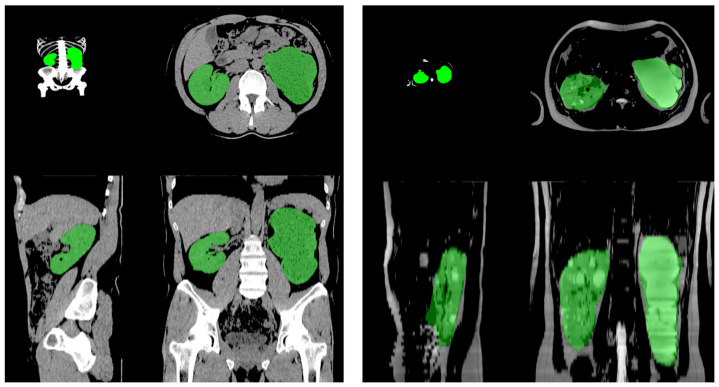
The CT scan and MRI imaging for total kidney volume calculation using AI-assisted auto-segmentation.

The workflow involves three steps: data preprocessing, ADPKD region segmentation, and post-processing. The final output is a 3D segmentation mask used to compute TKV. Additionally, we sought to compare the performances of two imaging modalities (CT vs. MRI). The MRI quality was not unified, and diverse phases and enhancement shots were applied. We chose T2-weighted images for clear kidney border demarcations and used the other phase if the T2-weighted images were unavailable (Figure 1). We utilized a convolutional neural network-based semantic segmentation model, specifically the 3D Dynamic U-Net architecture, to automatically calculate TKV from abdominal imaging. The model was trained on a multi-modality dataset consisting of paired abdominopelvic CT and T2-weighted MRI scans with voxel-wise annotated masks from patients with ADPKD. The original data set was acquired from seven hospitals using eight different types of CT machines within various institutions. The 753 ADPKD patient cases, comprising 95,117 images, were used to develop the algorithm. In total, 38,261 hand-labeled segmentation image slices by three independent expert radiologists were used for training set. The rest of the images were divided into validation and test sets. For MRI inputs, axial T2-weighted sequences were prioritized due to their superior kidney border demarcation; alternative phases were used only when T2-weighted images were unavailable. For CT, the model demonstrated robust performance across the various axes of image slides, including both contrast-enhanced and non-contrast CT scans. All CT and MRI scans were acquired in DICOM format and underwent preprocessing that included voxel resampling to a standard resolution (typically 1.5 × 1.5 × 3.0 mm^3^) and intensity normalization to ensure consistency across institutions and modalities. The segmentation pipeline includes three steps: (1) image preprocessing, (2) semantic segmentation of kidneys and cystic regions, and (3) post-processing to compute TKV. The final output is a binarized 3D probability mask, which is used to derive TKV by summing the segmented voxels. This model demonstrated robust performance across both CT and MRI modalities, achieving a Dice similarity coefficient of 0.979 (95% CI, 0.966–0.990) [19].

In this study, the algorithm progressively improved the advanced segmentation. While the current study applies the algorithm exclusively to CT datasets, its cross-modality design allows future application to MRI without architectural modification. This study was approved by the Institutional Review Board (Number: 2024-05-020-003) of the Sungkyunkwan University Medical College Kangbuk Samsung Hospital.

### 2.2. Statistical Analysis

Patients’ baseline characteristics were described as median with minimal to maximum values due to skewed distribution with Student’s *t*-test and chi-square test. All data were evaluated as having a non-Gaussian distribution. Comparisons were made using the intraclass correlation coefficient (ICC), Bland–Altman plots with nonparametric Spearman’s correlation, with GraphPad Prism 10.4.1 (Boston, MA, USA), and MedCalc^®^ version 23.1.3 (MedCalc Software Ltd., Ostend, Belgium). All reported *p*-values are two-tailed and considered statistically significant at <0.05.

## 3. Results

### 3.1. Patient Characteristics

During the study period, a total of 32 participants were identified. The median age was 56 years, ranging from 31 to 95 years old, with a median creatinine level of 1.16 mg/dL and a total kidney volume of 1200.24 mL. The majority of cases fall into categories 1B and 1C, indicating intermediate progression. Category 1E has one case, representing the most advanced stage of the disease (Table 1).

### 3.2. Correlation Analysis

**Figure 2:**
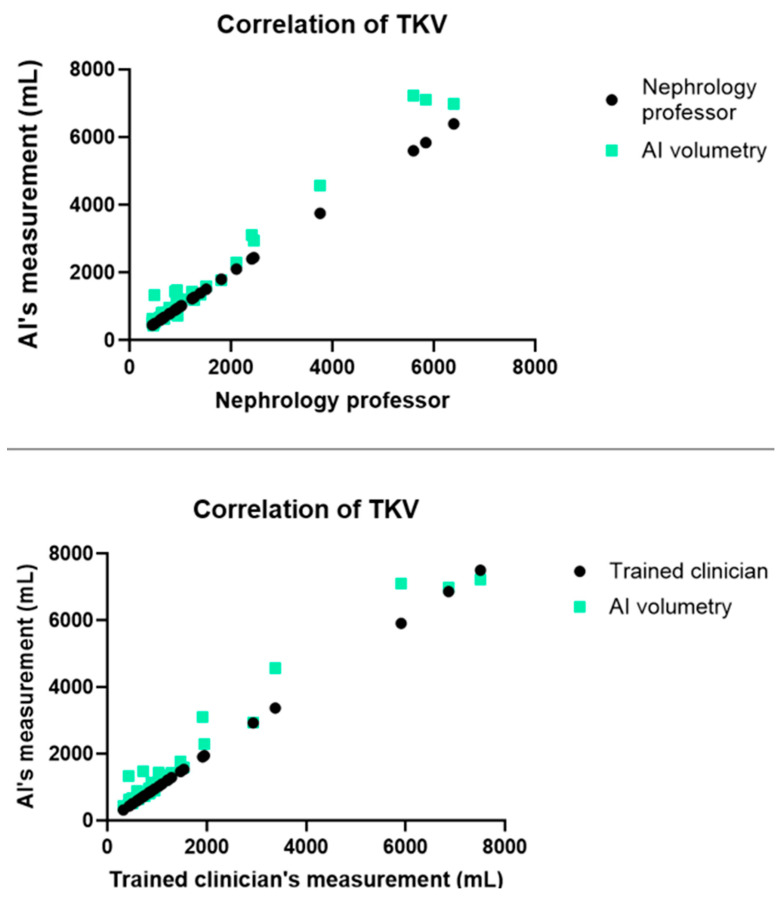
Interclass correlation coefficients (ICCs) between human-sourced tracing volumetry and artificial intelligence (AI) auto-segmentation using computed tomography and magnetic resonance imaging.

AI-based kidney volumetry using CT showed a high ICC compared with that of the manual ellipsoid method (compared with nephrology professor ICC: 0.991, 95% CI = 0.9780–0.9948, *p* < 0.01; compared with well-trained clinician ICC: 0.983, 95% CI = 0.9608–0.9907, *p* < 0.01) (Figure 2). AI-based kidney volumetry and human manual measurement showed high concordance.

### 3.3. Performance of the AI-Based Volumetry

**Figure 3:**
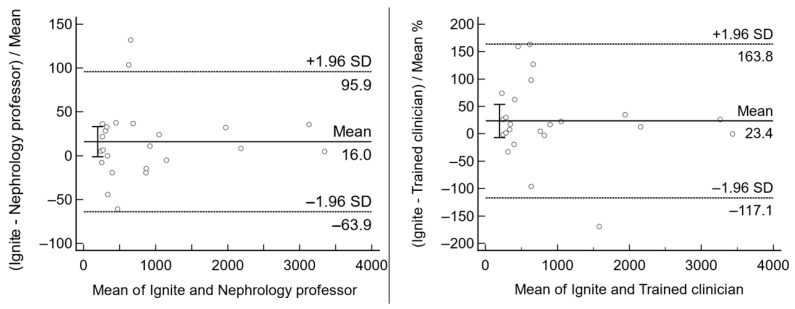
Absolute volume agreement between the human-sourced ellipsoid method vs. AI volumetry.

The degree of concordance between volumetric measurements obtained using the traditional, human-performed ellipsoid method and those generated by AI-based volumetric analysis was evaluated. We compared the clinical relevance between the ellipsoid method by human resource vs. AI volumetry using Bland–Altman plots (Figure 3). Two human-sourced ellipsoid methods showed no differences (absolute arithmetic mean difference = −25.4 mL, 95% CI = −171.0425 to 120.3180, *p* = 0.72); however, the AI volumetry values were significantly larger than those of the trained clinician (+260.7 mL, 95% CI = 124.8499–396.5385, *p* < 0.01). AI volumetry and a nephrology professor’s results were comparable (+159.5 mL, 95% CI = −11.8368 to 330.7818, *p* = 0.07). In a clinical setting, the MICs of AI volumetry and human-sourced methods were similar (Table 2). Notably, the results of the AI were largely similar to the professor’s opinion, particularly in distinguishing between 1B and 1C.

## 4. Discussion

These findings indicate that AI volumetry not only improves measurement accuracy but also reduces variability and bias inherent to manual ellipsoid estimations. We compared TKV measurements obtained from CT in patients with ADPKD using the manual ellipsoid method versus an AI-based volumetry approach and evaluated the agreement and potential error between the two methods. The AI demonstrated excellent agreement with the ellipsoid method and outperformed the trained clinician regarding measurement accuracy. In terms of MIC classification, the AI and ellipsoid methods exhibited similar distribution patterns.

The MIC facilitates TKV measurement; however, it has some limitations. The semi-quantitative nature of this method introduces inherent errors, including variability from human judgment. Accurate measurements require clinicians to have a thorough understanding of renal anatomy, imaging principles, and appropriate techniques for defining kidney boundaries. Additionally, consistent imaging quality is essential to ensure reliable results. For example, clinicians need to carefully select the appropriate slices for measurement to avoid inaccuracies caused by poor image resolution or suboptimal sectioning of the kidney. Consequently, proper training and expertise are essential for clinicians using this method.

Despite these advancements, kidney volume remains the biomarker and predictive model for ADPKD progression. The reliance on the TKV as a surrogate marker underscores the urgency for more accurate, efficient, and accessible measurement techniques. Improving the precision and usability of kidney size assessments will enhance the prediction of disease progression and optimize the selection of patients for tolvaptan therapy. Future innovations in imaging technologies and predictive models are essential to refining ADPKD management and ensuring better outcomes for affected individuals.

AI-supported kidney volume measurement tools are emerging. Kline et al. reported automated segmentation methods using MRI data from 20 patients with ADPKD to validate the automated algorithm against manual measurements. The agreement was assessed using Bland–Altman analysis and linear correlation [15]. Gastel et al. trained a deep learning-based segmentation model, validated data from 440 patients, and further tested an independent dataset of 100 patients using MRI [18]. The automated measurements demonstrated precision comparable to manual tracing (ICC: TKV 0.998), with 98% agreement in Mayo risk classification. This automated approach offers a reliable and efficient tool for assessing disease progression and treatment efficacy in patients with ADPKD. Shin et al. also developed and validated an AI-based 3D volumetry model for calculating the TKV in 753 patients with ADPKD [19]. Using a deep-learning framework, 3D Dynamic U-Net, the model was also implemented into a software-as-a-service to support tolvaptan prescription decisions. The AI-based TKV measurement system demonstrated expert-level accuracy (Dice score achieved = 0.979), offering a reliable and efficient tool for monitoring disease progression and therapeutic outcomes in ADPKD. It is a significant tool with notable consistency in clinical settings, reducing time and labor. Park et al. assessed the applicability of the MIC in predicting renal outcomes for Korean patients with ADPKD [20]. They reported that the MIC effectively classified disease progression rates based on TKV and age, demonstrating utility in predicting rapid progressors among Korean patients. While the Higashihara equation offered more stable growth rate estimations, it was less effective in predicting renal outcomes than MIC [21]. Thus, the MIC remains a reliable predictive tool for Korean patients with ADPKD, implying the need to develop more efficacious and accurate approaches for measuring kidney volume.

As pharmacological treatment is considered to begin with rapid disease progression, such as the MIC 1C and higher Predicting Renal Outcome in Polycystic Kidney Disease (PROPKD) score [3]. The higher PROPKD score points to a greater risk of progression to ESKD. The MIC and PROPKD should not be interchangeable, so patient evaluation and monitoring need consistency. MICs are generally preferred, and genotypic and/or phenotypic information play an additional role [22]. Metabolomics and sodium measurement in cyst fluid are being explored for deeper phenotyping and understanding disease mechanisms, but these are not yet standard clinical tools [23]. In particular, accurately distinguishing between MIC 1B and 1C is crucial. Additionally, in cases classified as 1E, the kidneys are excessively large; therefore, it is essential to demarcate the kidney in relation to the liver margin. During the concurrent counting of the number of MIC 1A and 1B, the ellipsoid method and AI volumetry showed similar group distributions. However, AI volumetry tended to classify more cases as MIC 1C than did the other evaluators. The distinction between Classes 1B and 1C has been previously highlighted as requiring careful consideration. Shi et al. examined the TKV agreement between manual segmentation and ellipsoid and found that the ICC was good between both methods [24]. Similarly, they suggested that a cautious approach is necessary with MIC 1B and 1C.

In contrast to the comparison with the results of the professor, AI-based volumetry-measured TKVs were 260.7 mL higher than those obtained by the trained clinician. This suggests that, as previously reported by Di Pietro et al., the overestimation in the ellipsoid method is largely owing to human error [17]. Such discrepancies raise concerns that non-expert measurements could result in undertreatment, particularly when TKV is underestimated. The ellipsoid method shows high variability between observers, particularly when used by less experienced operators. This may be particularly unfavorable when TKV is used as a biomarker for longitudinal follow-up and assessment of drug efficacy through repeated measurements. Demoulin et al. reported that the estimated TKV using the ellipsoid equation in patients with ADPKD showed poor repeatability and reproducibility compared with that of manual TKV [25].

This study has some limitations. First, this study was conducted in a retrospective manner using data from a small sample size from a single-center Asian population and did not include a comparison with manual kidney segmentation (stereology). Second, the detailed algorithmic development process was not described, as it was not the primary focus of this study. Detailed information can be found in the original articles by the authors of this study [18]. Third, owing to data security and personal information protection regulations, the original training data used for algorithm development cannot be shared. However, selective access to the measurement results may be granted upon request. Finally, we only used CT scans, and the results do not reflect analyses based on our ultrasound data. Considering the differences in imaging modalities, the results should be interpreted with caution. In addition, we did not have access to the patient’s genetic data set. Furthermore, we did not see if we could predict the progression of the disease on a continuous basis.

We evaluated the performance of an AI-based volumetry tool for measuring TKV in patients with ADPKD using CT data from a single-center Asian cohort. The AI demonstrated excellent agreement with the professional manual ellipsoid method concerning TKV measurement and showed greater accuracy than did a trained clinician. This AI-assisted algorithm was developed using multicenter images with diverse CT manufacturers. Therefore, it could be a useful assisting tool regardless of CT machines and contrast enhancement. While the ellipsoid method remains common owing to its accessibility, it is subject to operator-dependent variability and may overestimate or underestimate TKV, particularly when performed by less experienced users. AI-based systems offer advantages in consistency, efficiency, and scalability, which are vital for longitudinal monitoring and therapeutic decision-making in ADPKD.

## Figures and Tables

**Table 1:** Characteristics of the 32 patients in this study.

Category (Numbers = 32)	Number or Median (min–max)
Sex (male)	18 (56.25%)
Age (years)	56 (31–95)
Heights (cm)	169 (152–186)
Weights (kg)	69 (45–109)
Creatinine (mg/dL)	1.16 (0.57–4.77)
Mayo imaging classification (MIC) (using the ellipsoid method, nephrology professor) (%)	
1A	4 (12.5%)
1B	10 (31.25%)
1C	12 (37.5%)
1D	5 (15.63%)
1E	1 (3.13%)
Total kidney volume (mL)	1200.24 (432.19–6984.2)

**Table 2:** MIC using the human-sourced ellipsoid method vs. AI volumetry.

MIC	Nephrology Professor *n*, (%)	AI Volumetry *n*, (%)	Trained Clinician *n*, (%)
1A	4 (12.5%)	3 (9.38%)	5 (15.6%)
1B	10 (31.25%)	10 (31.25%)	8 (25%)
1C	12 (37.5%)	16 (50%)	10 (31.25%)
1D	5 (15.63%)	1 (3.13%)	7 (21.88%)
1E	1 (3.13%)	2 (6.25%)	2 (6.25%)

## Data Availability

Selective access to the measurement results may be granted upon request.

## Abbreviations

The following abbreviations are used in this manuscript:

MIC	Mayo imaging classification
TKV	total kidney volume
PKD	polycystic kidney disease
MRI	magnetic resonance imaging
CT	computed tomography
ICC	intraclass correlation coefficient
ADPKD	autosomal dominant polycystic kidney disease
htTKV	height-adjusted total kidney volume
